# Percutaneous Ultrasound-Guided Ablation of Implanted Parathyroid Glands for Recurrent Hyperparathyroidism After Total Parathyroidectomy

**DOI:** 10.3390/jcm15135211

**Published:** 2026-07-03

**Authors:** Guanqi Hang, Jiunn Wong, Sum Leong, Julia Andres, Weiyong Lee, Chow Wei Too

**Affiliations:** 1 Department of Vascular and Interventional Radiology, Singapore General Hospital, Singapore 169608, Singapore; 2 Department of Renal Medicine, Singapore General Hospital, Singapore 169608, Singapore

**Keywords:** ultrasound, hyperparathyroidism, radiofrequency ablation, cryotherapy, microwave ablation, total parathyroidectomy with autotransplantation, safety

## Abstract

**Purpose:** Tertiary hyperparathyroidism is a frequent complication of end-stage kidney disease that may require parathyroidectomy when medical therapy fails. Recurrent hyperparathyroidism may occur after total parathyroidectomy with autotransplantation. This study assessed the safety, feasibility, and efficacy of percutaneous ultrasound-guided ablation for implanted parathyroid glands in patients with recurrent disease after total parathyroidectomy with autotransplantation. **Materials and Methods:** Eleven patients with recurrent hyperparathyroidism following total parathyroidectomy with autotransplantation who underwent percutaneous ultrasound-guided ablation were retrospectively reviewed. Pre- and post-procedure serum intact parathyroid hormone, calcium, phosphate, and alkaline phosphatase levels were extracted from electronic medical records and analyzed. Ablation modalities used in the study included radiofrequency ablation, microwave ablation, and cryoablation. Procedure-related complications were recorded. Patients were followed for 12 months with serial biomarker monitoring. Technical success was defined as ≥80% ablation of implanted parathyroid glands, and clinical success as meeting at least two of the following: (1) >50% intact parathyroid hormone reduction, (2) correction of hypercalcemia, (3) discontinuation of calcimimetic therapy. **Results:** Technical success was achieved in 10 of 11 (90.9%) patients; one patient had incomplete ablation due to proximity of the gland to the skin. Among technically successful cases, all (100%) achieved clinical success, showing marked decreases in intact parathyroid hormone, normalization of calcium and phosphate, and reduced calcimimetic use. Transient hypocalcemia occurred in six patients, five requiring intravenous calcium replacement. No long-term complications were observed. **Conclusions:** Percutaneous ultrasound-guided ablation of implanted parathyroid glands is a safe and effective option for managing recurrent hyperparathyroidism, offering a viable alternative to surgery.

## 1. Introduction

Tertiary hyperparathyroidism is a common condition in end-stage kidney disease (ESKD) patients who have undergone dialysis for an extended period [1,2,3,4]. The condition is characterized by autonomous overproduction of parathyroid hormone that is resistant to medical therapy [3,5]. It is important to acknowledge that some authors reserve the term “tertiary hyperparathyroidism” for persistent autonomous hyperparathyroidism that arises subsequent to successful renal transplantation [6]. In this study, however, the term is used more broadly to denote autonomous, refractory parathyroid overactivity in patients undergoing prolonged dialysis, a usage consistent with its application in several published series involving percutaneous ablation for renal hyperparathyroidism [7,8]. Hyperparathyroidism manifests through a range of systemic symptoms driven by excess parathyroid hormone and hypercalcemia, with early disease often presenting as asymptomatic or with nonspecific complaints such as fatigue, muscle weakness, and neurocognitive or mood changes [9,10]. As the condition progresses, skeletal involvement may lead to bone pain, reduced bone mineral density, and increased fracture risk, while renal complications commonly include nephrolithiasis, polyuria, and polydipsia [9,10]. Gastrointestinal symptoms such as constipation, nausea, and abdominal discomfort are also frequently reported, reflecting the multisystem impact of sustained calcium dysregulation [9,10].

For patients who have failed medical therapy, the Kidney Disease: Improving Global Outcomes (KDIGO) guideline recommends parathyroidectomy [11]. Parathyroidectomy can be performed as subtotal parathyroidectomy, total parathyroidectomy with autotransplantation (usually in the deltoid), or total parathyroidectomy without autotransplantation. Each approach balances the competing risks of recurrent hyperparathyroidism and permanent hypoparathyroidism.

Because kidney transplantation often involves prolonged waiting times, and subtotal parathyroidectomy carries a risk of recurrent hyperparathyroidism, our center primarily performs total parathyroidectomy with autotransplantation, as described by Ng et al. [12], with glands typically implanted in the deltoid region. Specifically, parathyroid tissue fragments are implanted into the subcutaneous fat overlying the deltoid muscle (subcutaneous implantation), rather than within the muscle substance itself. This approach facilitates autograft recovery and improves graft visibility during postoperative ultrasound surveillance and, if required, for percutaneous ablation access [12]. Conventionally, recurrent hyperplasia of the deltoid autograft is managed surgically; however, repeat surgery has disadvantages. Complete excision risks permanent hypoparathyroidism, the small implants can be difficult to identify (especially after dissection distorts tissue planes), and many patients with end-stage kidney disease are poor candidates for general anesthesia.

Ultrasound-guided percutaneous ablation is well established for treating lesions in the liver and thyroid, and has also been applied to native parathyroid glands. When performed for implanted parathyroid autografts, it enables selective partial ablation (rather than complete removal), potentially reducing the risk of permanent hypoparathyroidism while avoiding general anesthesia. The present study evaluated the safety, feasibility, and efficacy of ultrasound-guided percutaneous ablation of implanted parathyroid glands in patients with recurrent hyperparathyroidism after total parathyroidectomy with autotransplantation.

## 2. Materials and Methods

### 2.1. Patient Characteristics

This retrospective study was conducted at a tertiary medical center and included consecutive patients with recurrent hyperparathyroidism after total parathyroidectomy with autotransplantation who underwent ultrasound-guided percutaneous ablation of implanted parathyroid tissue. Patients were referred to interventional radiology when they were unsuitable for, or unwilling to undergo, repeat surgery. Procedures were performed between May 2020 and July 2022.

Baseline demographic characteristics, medications, procedural details, and serum biomarkers (intact parathyroid hormone, calcium, phosphate, and alkaline phosphatase) were extracted from electronic medical records and analyzed. The study protocol was reviewed and approved by our institution’s Institutional Review Board (IRB reference number: 2022/2647) and conducted in accordance with the Declaration of Helsinki. Written informed consent was obtained from each patient before the procedure.

### 2.2. Definition of Recurrent Hyperparathyroidism

Recurrent hyperparathyroidism was diagnosed using a combination of biochemical findings and treatment requirements after prior parathyroidectomy: persistently elevated intact parathyroid hormone with hypercalcemia attributable to hyperparathyroidism, and/or the need for high-dose calcimimetic therapy (e.g., cinacalcet) with activated vitamin D to maintain biochemical control.

### 2.3. Inclusion and Exclusion Criteria

Inclusion criteria were: (1) ESKD patients on renal replacement therapy; (2) prior total parathyroidectomy with autotransplantation; (3) recurrent hyperparathyroidism refractory to medical therapy; (4) sestamibi scintigraphy localizing hyperfunctioning parathyroid tissue to the deltoid autograft; and (5) sonographic visualization of a hypertrophied implanted parathyroid gland suitable for percutaneous ablation.

Exclusion criteria were: (1) uncorrectable coagulopathy; (2) refusal of percutaneous ablation; (3) inability to cooperate with the procedure; and (4) functioning renal allograft after kidney transplantation.

### 2.4. Pre-Ablation Assessment

Patients who failed medical therapy for recurrent hyperparathyroidism were referred for interventional radiology assessment after sestamibi scintigraphy localized hyperfunctioning parathyroid tissue to the deltoid autograft. The pre-procedural ultrasound examination focused on identifying the hypertrophied implanted gland most consistent with the clinical and biochemical findings; a systematic survey to enumerate all implanted tissue was not routinely performed. Ultrasound was also used to assess depth from the skin and proximity to critical structures to determine feasibility (including whether hydrodissection could be performed).

Serum intact parathyroid hormone, calcium, phosphate, and alkaline phosphatase were tested within 24 h before the procedure. Coagulation parameters were checked to confirm procedural safety.

### 2.5. Ablation Techniques

Ultrasound-guided percutaneous ablation was performed in an inpatient setting to allow close monitoring and management of post-procedural calcium and phosphate disturbances. Immediately before ablation, ultrasound was used to confirm the location and depth of the target gland. The skin was prepared using standard aseptic technique. Local anesthesia (1% lidocaine and 0.5% bupivacaine) was administered, and saline was injected subcutaneously when needed to increase the skin-to-target distance and provide thermal insulation. The planned ablation target was partial gland ablation (approximately 80–90%) to balance biochemical control against the risk of permanent hypoparathyroidism; the extent of ablation was assessed by intra-procedural ultrasound appearance rather than formal volumetric calculation.

All ultrasound-guided percutaneous ablations were performed by two senior authors (CWT and SL), each with more than 10 years of experience in ultrasound-guided ablation procedures. Ablation modality included radiofrequency ablation (RFA) (17G STARmed radiofrequency ablation probe; STARmed Co., Ltd., Goyang, Republic of Korea), microwave ablation (MWA) (16G ECO microwave ablation probe; Nanjing ECO Medical Technology Co., Ltd., Nanjing, China), and cryoablation (IceRod 1.5 mm × 175 mm probe; Galil Medical Inc., Saint Paul, MN, USA).

**Figure 1:**
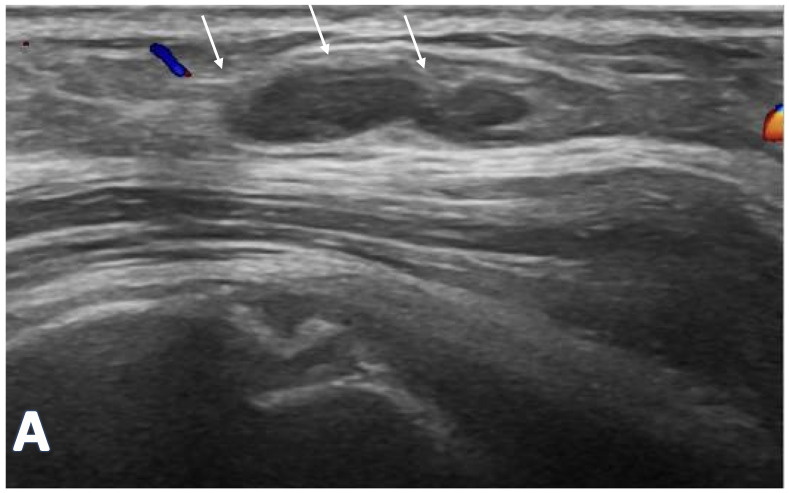

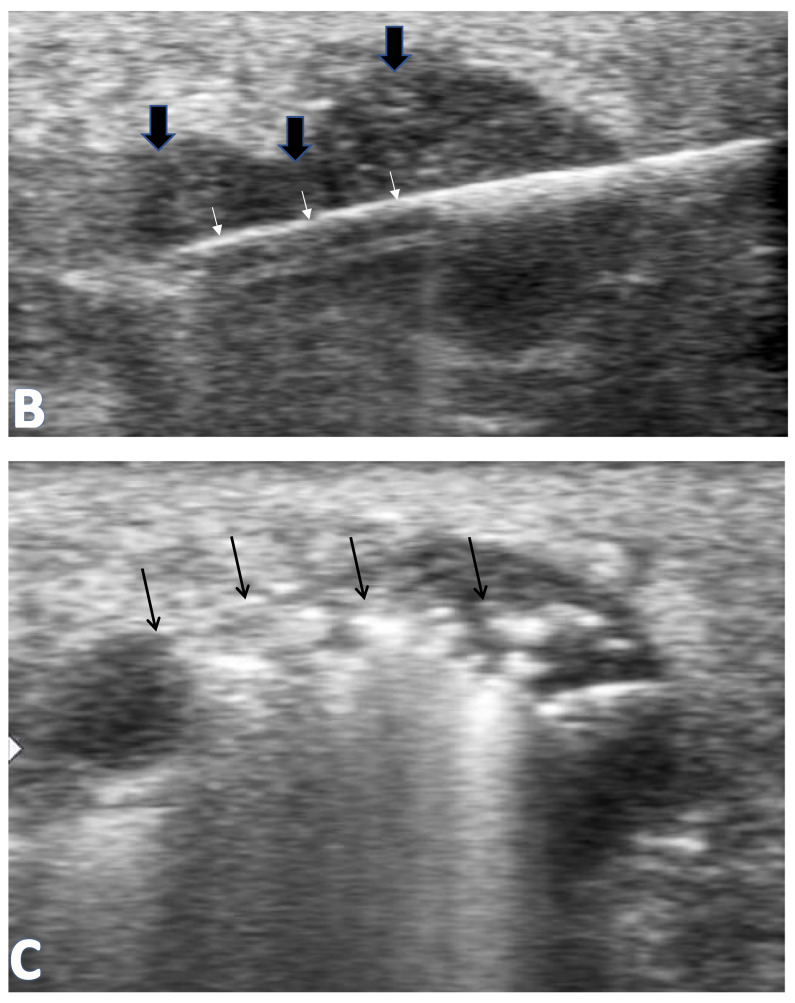
Ultrasound characteristics of a patient‘s deltoid parathyroid autoimplant (male, 66 years old, on hemodialysis for 8 years) before and after microwave ablation (MWA). (**A**) Ultrasound of the parathyroid autoimplant in the deltoid muscle before ablation: a 5 × 3 cm hypoechoic nodule is the hyperplastic parathyroid autoimplant (white arrow). (**B**) Ablation probe (white arrow) was inserted into the parathyroid autoimplant (bolded black arrow). (**C**) Post-ablation echogenic clouds were generated in the parathyroid autoimplant (black arrow).

**Figure 2:**
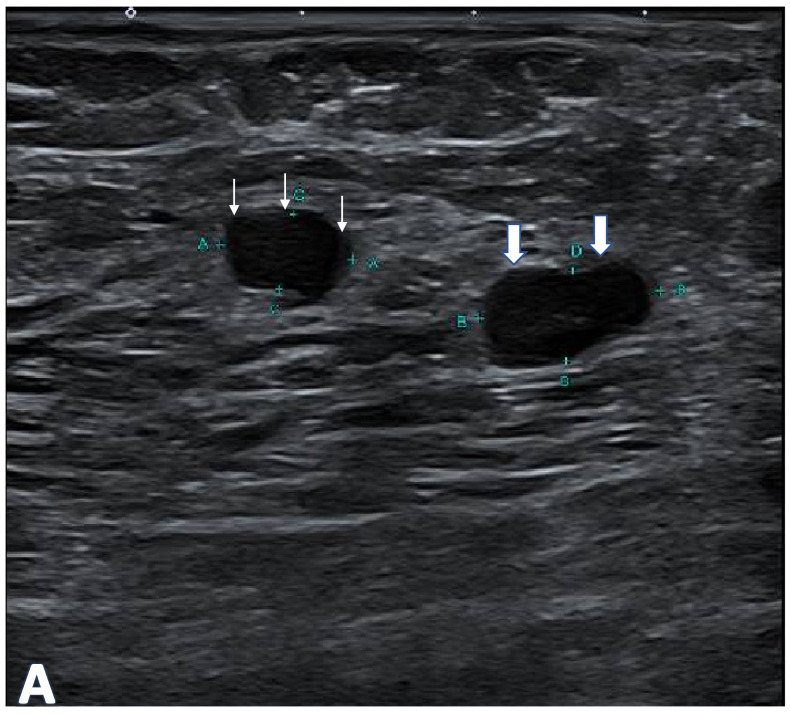

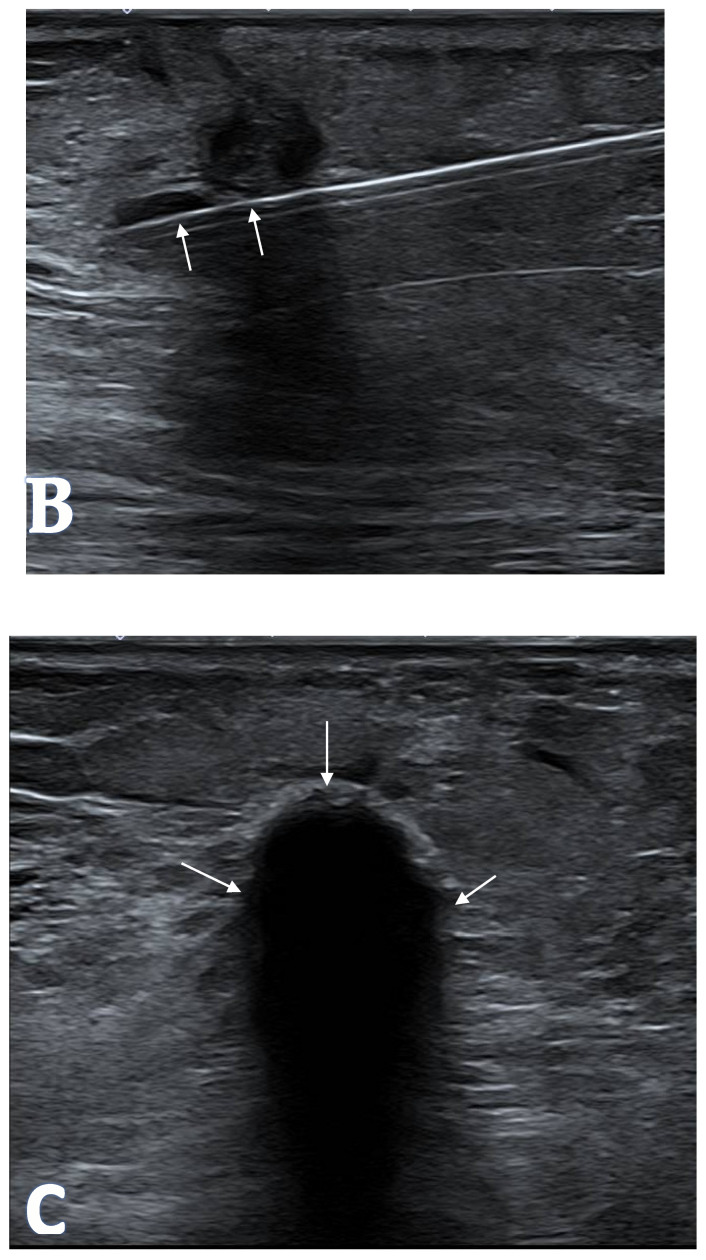
Ultrasound characteristics of a patient’s deltoid parathyroid autoimplant (male, 68 years old, on hemodialysis for 10 years) before and after cryotherapy (CA). (**A**) A 3.5 × 2.5 cm hypoechoic parathyroid autoimplant in the deltoid muscle before cryotherapy: normal parathyroid autoimplant on the left (white arrow) and hyperplastic parathyroid autoimplant on the right (bolded white arrow). (**B**) Cryo probe (white arrow) was inserted in the hypoechoic parathyroid autoimplant. (**C**) Hypoechoic “ice ball” (white arrow) was formed inside the parathyroid gland.

The size of the active tip and type of probes were selected based on the size of the glands. For RFA and MWA, the glands were ablated using the “moving shot technique” proposed by Baek et al. [13,14] (Figure 1). The moving shot technique is a probe-manipulation strategy in percutaneous thermal ablation in which the active tip of the electrode is intentionally and sequentially repositioned within the target lesion. Ablation energy is delivered in short, controlled applications at multiple adjacent locations, typically beginning in the deepest portion of the lesion and progressing toward the superficial margin. These overlapping ablation zones coalesce into a continuous, conformal necrotic volume, enabling precise control of ablation extent while avoiding excessive heating from a single stationary application. This technique is particularly suited for small or irregularly shaped lesions, where accurate spatial coverage is required [13,15]. Figure 1 and Figure 2 illustrate the MWA and cryotherapy of the deltoid parathyroid autoimplants.

In the same setting, a central venous catheter was inserted into the right internal jugular vein to facilitate blood sampling after the procedure and calcium replacement.

### 2.6. Follow-Up and Collection of Clinical Data

Immediate post-procedure complications were assessed, including arm pain, numbness, bleeding, and skin thermal injury. Serum intact parathyroid hormone, calcium, and phosphate were measured at 1 day, 3 days, 7 days, and at 1, 3, 6, and 12 months after the procedure. Serum alkaline phosphatase was measured at 1, 3, 6, and 12 months.

### 2.7. Definition of Successful Procedure (Technical and Clinical)

Technical success was defined as achieving the planned partial ablation coverage of the target implanted parathyroid gland (approximately 80% by intra-procedural ultrasound assessment), without premature termination due to safety concerns (e.g., inability to protect the skin).

Clinical success was defined as meeting at least two of the following criteria over follow-up: (1) >50% reduction in intact parathyroid hormone compared with baseline; (2) correction of hypercalcemia; and (3) discontinuation of calcimimetic therapy. Biomarker assessments were performed at the predefined follow-up time points.

### 2.8. Statistical Analysis

Statistical analyses were performed using Stata (version 17; StataCorp LLC, College Station, TX, USA). Continuous variables are presented as mean ± standard deviation. Changes from baseline were assessed using paired *t*-tests. A two-sided *p*-value < 0.05 was considered statistically significant.

## 3. Results

### 3.1. Patient Demographics and Technical Success

Patient demographics are summarized in Table 1. Eleven patients with recurrent hyperparathyroidism were included (6 men [54.5%] and 5 women [45.5%]); mean age was 60.4 ± 8.2 years. All patients had end-stage kidney disease and were receiving renal replacement therapy (hemodialysis, *n* = 10 [90.9%]; peritoneal dialysis, n = 1 [9.1%]). Mean duration of dialysis was 16.0 ± 5.2 years. Pre-procedure mean intact parathyroid hormone level was 187.0 ± 83 pmol/L (normal range 0.9–6.2 pmol/L), serum calcium was 2.5 ± 0.3 mmol/L (normal range 2.09–2.46 mmol/L), phosphate was 1.8 ± 0.5 mmol/L (normal range 0.9–1.5 mmol/L), and alkaline phosphatase was 453.7 ± 360.6 U/L (normal range 39–99 U/L).

Technical success was achieved in 10 of 11 patients (90.9%). In the single technical failure, the target gland was too superficial to safely achieve the planned ablation coverage despite attempted hydrodissection, and the patient subsequently defaulted follow-up. Five patients (45.5%) underwent radiofrequency ablation, five (45.5%) underwent microwave ablation, and one (9.1%) underwent cryoablation. Radiofrequency ablation power ranged from 50 to 70 W, and microwave ablation power ranged from 10 to 20 W. Planned ablation coverage was approximately 80% of the target gland based on intra-procedural visual estimation on ultrasound.

Post-ablation hypocalcemia was noted in 6 (54.5%) patients, 5 (45.4%) of whom required intravenous calcium replacement. No long-term complications were observed in those patients during follow-up.

### 3.2. Post-Procedure Biochemistry Trends

**Figure 3:**
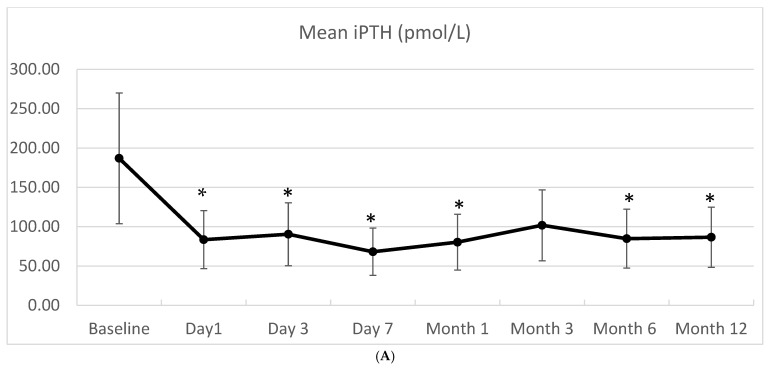

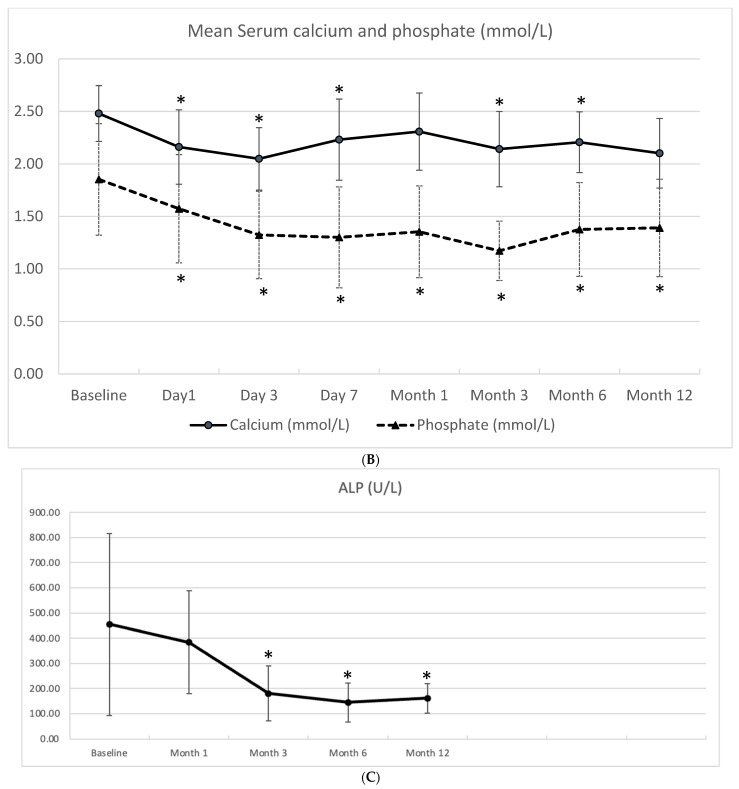
(**A**): serum iPTH trend pre- and post-ablation; (**B**): Serum calcium and phosphate trend; (**C**): Serum ALP trend. “*” indicates the value is statistically significantly different from the pre-procedure value. The vertical bar represents the standard deviation.

After the procedure, serum iPTH, calcium, phosphate, and ALP were measured on post-procedure day 1, day 3, day 7, month 1, month 3, month 6, month 12 (Table 2). The post-ablation serum biochemical trend is shown relative to baseline (Figure 3). Of 11 patients, 10 completed the 1-year follow-up.

Table 2 summarizes serum biochemical parameters at baseline and follow-up time points.

Significant reduction in serum iPTH was observed on post-procedure day 1 (*p* = 0.005), day 3 (*p* = 0.02), day 7 (*p* = 0.001), month 1 (*p* = 0.01), month 6 (*p* = 0.04), and month 12 (*p* < 0.01). In addition, there was a significant reduction in serum calcium on post-procedure day 1 (*p* < 0.01), day 3 (*p* < 0.01), day 7 (*p* < 0.01), month 3 (*p* < 0.01), and month 6 (*p* = 0.03). For serum phosphate, significant reduction was observed on day 1 (*p* = 0.01), day 3 (<0.01), day 7 (<0.01), month 1 (<0.01), month 3 (<0.01), month 6 (*p* = 0.03), and month 12 (*p* < 0.01). Last but not least, a significant reduction in serum ALP was also observed at 3 months (*p* = 0.02), 6 months (*p* = 0.04), and 12 months (*p* = 0.02) post-procedure (Figure 3).

### 3.3. Clinical Success Rates

Clinical success was achieved in 10 of 10 (100%) patients with technically successful ablation. At 12 months, all 10 patients achieved a >50% reduction in intact parathyroid hormone and maintained calcium and phosphate values within the institutional reference ranges; three patients discontinued calcimimetic therapy. The remaining seven patients continued calcimimetic therapy at reduced doses; complete discontinuation was not pursued because intact parathyroid hormone remained above the target range or because the treating nephrologist opted to maintain low-dose calcimimetic therapy as prophylaxis against biochemical recurrence. As calcimimetic discontinuation was only one of three criteria required for clinical success, its partial attainment in this cohort did not preclude classification as clinical success.

## 4. Discussion

This study suggests that ultrasound-guided percutaneous ablation of implanted parathyroid glands is a safe and effective option that avoids the need for general anesthesia. Treatment was associated with a marked reduction in intact parathyroid hormone, normalization of calcium and phosphate, and decreased calcimimetic use.

The advantages of ultrasound-guided percutaneous ablation include its minimally invasive nature, lower risk of complications, and rapid recovery. Furthermore, ultrasound guidance during the procedure helps ensure accurate targeting of the implants. It allows selective partial gland ablation, reducing the risk of injury to surrounding structures, such as the skin and subcutaneous tissue, and potentially reducing the risk of permanent hypoparathyroidism. Moreover, no significant complications were reported in our study, and all patients tolerated the procedure well, which further supports the safety of percutaneous ablation for the treatment of recurrent hyperparathyroidism. This procedure is potentially repeatable.

This study, together with previous studies reporting successful outcomes of percutaneous ablation for native parathyroid glands [16,17,18,19], will hopefully expand the role of percutaneous treatment in the management of hyperparathyroidism.

Although studies comparing surgical removal and percutaneous ablation of implanted parathyroid glands are scarce, a meta-analysis of 15 studies and 1115 participants found that both percutaneous ablation and surgery of native parathyroid glands are effective modalities for treating hyperparathyroidism [20]. Percutaneous ablation has been shown to reduce the risk of hoarseness and hypocalcemia [20].

For recurrent hyperparathyroidism arising from deltoid autografts, repeat surgical exploration and partial or complete excision can achieve biochemical control, but it is technically challenging because grafts are small and tissue planes may be distorted by prior surgery. Complete removal increases the risk of permanent hypoparathyroidism, while incomplete excision may lead to persistent or recurrent disease. In contrast, ultrasound-guided percutaneous ablation enables image-guided targeting and selective partial ablation under local anesthesia, which may be advantageous in patients with significant comorbidity. Although surgical re-exploration can achieve precise anatomical localization under direct vision, ultrasound-guided percutaneous ablation offers comparable lesion targeting in the subcutaneous deltoid region, where the implants are superficial and consistently amenable to sonographic visualization.

Limitations include the retrospective design, small cohort size, and use of multiple ablation modalities, which precluded meaningful comparison between techniques. Post-procedural calcium supplementation (including intravenous calcium replacement in some patients) may have influenced early calcium trends and limits the interpretation of calcium normalization as an isolated endpoint. In addition, follow-up was limited to 12 months; therefore, long-term recurrence risk could not be evaluated.

The choice of ablation modality was based on operator preference rather than a pre-specified lesion-based protocol, and individual lesion dimensions stratified by ablation type were not prospectively recorded. These factors preclude any comparison of efficacy between techniques and represent an important consideration for the design of future studies.

Complete data on the interval between the index parathyroidectomy with deltoid autoimplantation and percutaneous ablation were not available for all patients, as some had undergone their original surgery at external institutions without accessible operative records. This precluded reliable reporting of this interval and further limited the retrospective design. Future prospective studies should systematically capture this information.

In addition, technical success relied on intra-procedural ultrasound estimation of ablation coverage by two experienced operators, which introduces subjectivity; formal volumetric assessment was not performed and future studies should consider incorporating three-dimensional volumetric or contrast-enhanced ultrasound evaluation to provide more objective confirmation of ablation extent. Furthermore, patients were selected only if they were unsuitable for or declined surgery, which introduces selection bias and limits generalizability of the findings to the broader population of patients with recurrent hyperparathyroidism. The absence of a comparator arm (either surgical re-exploration or continued pharmacological therapy) prevents conclusions about the relative efficacy of percutaneous ablation compared with alternative management strategies.

## 5. Conclusions

In conclusion, this study provides valuable evidence on the safety and efficacy of ultrasound-guided percutaneous ablation for the treatment of recurrent hyperparathyroidism after total parathyroidectomy with autotransplantation. The study findings suggest that percutaneous ablation is a feasible and effective alternative treatment option for patients with recurrent hyperparathyroidism who are not suitable or willing to undergo surgery. Further large-scale, multicenter studies are needed to determine whether this procedure can become the first choice for the treatment of recurrent hyperparathyroidism after total parathyroidectomy with autotransplantation.

## Figures and Tables

**Table 1:** Summarizes patient characteristics and baseline biochemical parameters.

Patient Number	Total (N = 11)
Age, mean (SD)	60.4 (8.2)
Gender, N (%)	
Female	5 (45.5%)
Male	6 (54.5%)
Ethnicity	
Chinese	9 (81.8%)
Malay	2 (18.2%)
Dialysis type, Number (%)	
Peritoneal dialysis	1 (9.1%)
Hemodialysis	10 (90.9%)
Duration of dialysis, mean (SD) (years)	16 (5.2)
Length of stay (LOS), median (SD) (days)	5.2 (6.1)
Baseline iPTH mean (SD) (pmol/L)	187.0 (83.0)
Baseline Ca, mean (SD) (mmol/L)	2.5 (0.3)
Baseline PO4, mean (SD) (mmol/L)	1.8 (0.5)
Baseline ALP, mean (SD) (U/L)	453.7 (360.6)
Incidence of post-ablation serum hypocalcemia, N (%)	6 (54.5%)
Requirement of intravenous replacement of calcium, Number of patients (%)	5 (45.4%)
Ablation type, Number (%)	
Microwave	5 (45.5%)
Cryotherapy	1 (9.1%)
Radiofrequency	5 (45.5%)
Ablation power, range (Watt)	
Microwave	10–20
Radiofrequency	50–70
Percentage of glands ablated, mean (SD) (%)	80.0 (6.1)
Successful cases and rate (%)	10 (90.9%)

**Table 2:** Biochemistry data before and after intervention.

Time	iPTH (pmol/L)	Calcium (mmol/L)	Phosphate (mmol/L)	ALP (U/L)
Pre-procedure	187.0 ± 83.0	2.5 ± 0.3	1.8 ± 0.5	453.7 ± 360.6
1 day	83.8 ± 36.8	2.2 ± 0.4	1.6 ± 0.5	NA
3 days	90.5 ± 40.0	2 ± 0.3	1.3 ± 0.4	NA
7 days	68.2 ± 30.0	2.2 ± 0.4	1.3 ±0.5	NA
1 month	80.4 ± 35.5	2.3 ± 0.4	1.4 ± 0.4	383.8 ± 204.2
3 months	101.8 ± 45.1	2.1 ± 0.4	1.2 ± 0.3	180.4 ± 108.8
6 months	84.8 ± 37.4	2.2 ± 0.3	1.4 ± 0.4	145.5 ± 77.3
12 months	86.7 ± 38.3	2.1 ± 0.3	1.4 ± 0.5	161.3 ± 58.2

Calcium normal range 2.09–2.46 mmol/L; iPTH normal range 0.9–6.2 pmol/L; phosphate normal range 0.9–1.5 mmol/L; ALP normal range 39–99 U/L. All values are expressed as mean ± SD.

## Data Availability

Restrictions apply to the datasets because the data are part of an ongoing study.
